# Namodenoson Inhibits the Growth of Pancreatic Carcinoma via Deregulation of the Wnt/β-catenin, NF-κB, and RAS Signaling Pathways

**DOI:** 10.3390/biom13111584

**Published:** 2023-10-27

**Authors:** Inbal Itzhak, Avital Bareket-Samish, Pnina Fishman

**Affiliations:** 1 Can-Fite BioPharma, Petah Tikva 49170, Israel; inbal@canfite.co.il; 2 BioInsight, Binyamina 3056814, Israel; avital@bioinsight-medcom.com

**Keywords:** A_3_AR agonist, apoptosis, mechanism of action, namodenoson, pancreatic cancer, RAS, Wnt/β-catenin

## Abstract

Namodenoson, an A_3_ adenosine receptor (A_3_AR) agonist, is currently being used in a phase III trial in advanced liver cancer. We examined the anti-growth effect of namodenoson on pancreatic carcinoma cells and investigated the molecular mechanism involved. BxPC-3 pancreatic carcinoma cells were cultured with namodenoson (5–20 nM for 24 h at 37 °C), and the Presto Blue assay was used to monitor cell growth. Western blot analyses were performed on BxPC-3 cells (20 nM namodenoson for 24 h at 37 °C) to evaluate the expression levels of cell growth regulatory proteins. In vivo studies involved the subcutaneous inoculation of BxPC-3 cells into nude mice, randomizing the mice into namodenoson (10 μg/kg twice daily for 35 days) vs. control, and monitoring tumor size twice weekly. Treatment with namodenoson was associated with the significant dose-dependent inhibition of BxPC-3 cell growth, which was mitigated by the A_3_AR antagonist MRS1523. Western blot analyses showed that namodenoson treatment modulated the expression of NF-κB, as well as proteins in the Wnt/β-catenin and the RAS signaling pathways, leading to the upregulation of apoptotic proteins (Bad, Bax). In vivo studies also showed the significant inhibition of pancreatic carcinoma tumor growth with namodenoson. In conclusion, our findings support the continued development of namodenoson as a treatment for pancreatic cancer.

## 1. Introduction

Pancreatic cancer is one of the most aggressive malignancies, with an overall 5-year survival rate of 8%. The primary cause of this low survival rate is the absence of early detection methods and the likelihood of early metastasis, resulting in a stage IV diagnosis in over half of cases (52%) [1]. Another key reason for the low survival rate is the limited effectiveness of the available treatment options, as the efficacy of gemcitabine alone or in combination with other chemotherapies is modest, and checkpoint inhibitors failed to show a significant clinical benefit [2]. Thus, novel and effective therapies for pancreatic cancer are clearly needed.

The Wnt/β-catenin and RAS signaling pathways have been identified as the main factors contributing to pancreatic carcinogenesis and resistance to therapy. These pathways were found to be involved in modulating a wide range of cellular processes, including differentiation, proliferation, motility, and downregulation of the apoptotic machinery [3,4]. Clinical research efforts aimed at developing therapies targeting these pathways are ongoing, but with limited success, primarily due to safety issues and insufficient efficacy/tumor resistance [3,5].

The A_3_ adenosine receptor (A_3_AR) is one of four receptors that mediate extracellular adenosine signaling [6]. Its mRNA and protein expression levels are upregulated in different tumor cell types (e.g., melanoma, prostate, colon, and hepatocellular carcinoma), but not in the adjacent normal tissues [7,8]. Namodenoson (CF102, Cl-IB-MECA), a synthetic ribose-based purine nucleoside, is a selective orally bioavailable A_3_ adenosine receptor agonist, whose mechanism of action has been shown to involve the deregulation of the NF-κB and the Wnt/β-catenin pathways, which leads to tumor cell apoptosis [9]. Phase I and II studies of namodenoson in advanced hepatocellular carcinoma (HCC) demonstrated excellent safety and efficacy in a subset of HCC patients with a Child–Pugh B score of 7 (CPB7) [10,11]. A pivotal phase III trial investigating namodenoson in HCC CPB7 patients is ongoing [12].

The current preclinical study examined the anti-growth effect of namodenoson on pancreatic carcinoma cells both in vitro and in vivo, and investigated the molecular mechanisms involved.

## 2. Materials and Methods

### 2.1. Reagents, Drug, and Cells

Dulbecco’s phosphate-buffered saline (PBS), RPMI medium, fetal bovine serum (FBS), RIPA buffer, and protease and phosphatase inhibitor cocktail (x100) were purchased from Thermo Fisher Scientific (Waltham, MA, USA). Dimethyl sulfoxide (DMSO) and MRS1523 were purchased from Sigma Chemical Co. (Rehovot, Israel). MRS1523 was dissolved in DMSO to yield a stock solution of 10 mM. Penicillin–streptomycin solution (x100) was purchased from IMBH (Beit Haemek, Israel).

Rabbit polyclonal antibodies against the phosphorylated-PKB/Akt (p-PKB/Akt), NF-κB, β-Catenin, A_3_AR, PI3K, GSK-3β, cyclin D1, ERK 1/2, MEK 1/2, Raf, Bad, and Bax were purchased from Santa Cruz Biotechnology Inc. (Dallas, TX, USA).

BxPC-3 (ATCC Cultures, Manassas, VA, USA), a cell line exhibiting epithelial morphology which was isolated from the pancreatic tissue of a 61-year-old female patient with pancreatic adenocarcinoma, was used for all the analyses. BxPC-3 (15 × 10^4^/mL) cells were grown in RPMI plus 10% FBS and 1x penicillin–streptomycin solution at 37 °C in a 5% CO_2_ incubator.

Namodenoson (2-chloro-N^6^-(3-iodobenzyl)-adenosine-5’-N-methyl-uronamide), lot No. 1884-050-05, was prepared by WuXi (Wuxi, China), and was stored at 4 °C in the dark. Namodenoson stock solution (10 mM) was prepared daily by dissolving the namodenoson powder in DMSO. For the in vitro studies, this solution was further diluted to a final concentration of 5, 10, and 20 nM in RPMI medium; for the in vivo studies, the namodenoson stock solution was diluted in PBS.

### 2.2. In Vitro Assays

For the tumor cell growth experiments, BxPC-3 cells (15 × 10^4^/mL) were incubated in the culture medium with control, 5, 10, or 20 nM namodenoson in 96-well microtiter plates for 24 h at 37 °C in a 5% CO_2_ incubator, after which cell growth inhibition was determined using the Presto Blue assay (Thermo Fisher Scientific, Waltham, MA, USA) used as per the manufacturer’s instructions. Experiments under the same conditions with 20 nM namodenoson were also conducted with and without 20 nM of the A_3_AR antagonist MRS1523 (diluted to the final concentration in RPMI medium).

Western blot analyses were performed using the same culture conditions as the Presto Blue experiments in 10 cm plates with 20 nM namodenoson or control. After incubation for 24 h at 37 °C in a 5% CO_2_ incubator, the cell samples were rinsed with ice-cold PBS and transferred to ice-cold RIPA buffer with 1x protease and phosphatase inhibitor cocktail for 20 min. Cell debris was removed using centrifugation for at 4 °C for 10 min, at 7500× *g*. The supernatant was utilized for the Western blot analyses. Protein concentrations were determined using the NanoDrop assay (ThermoFisher Scientific, Waltham, MA, USA). Equal amounts of the sample (50 μg) were separated by SDS-PAGE, using 12% polyacrylamide gels. The resolved proteins were then electroblotted onto nitrocellulose membranes (Schleicher & Schuell, Keene, NH, USA). Membranes were blocked with 1% bovine serum albumin and incubated with the relevant primary antibody (dilution 1:1000) for 24 h at 4 °C. Blots were then washed and incubated with the secondary antibody for 1 h at room temperature. Bands were recorded using BCIP/NBT color development kit (Promega, Madison, WI, USA).

### 2.3. In Vivo Assays

The in vivo experiments were performed in accordance with the guidelines established by the Institutional Animal Care and Use Committee at Can-Fite BioPharma (Petah Tikva, Israel).

Male nude Balb/C mice (Harlan Laboratories, Jerusalem, Israel), aged 2 months (mean weight, 25 g), received a subcutaneous flank injection of BxPC-3 cells (2.5 × 10^6^). The mice were maintained on a standardized pelleted diet and supplied with tap water. At Day 22 (a tumor size of 150–200 mm^3^), the animals were randomly assigned to two groups each containing 10 animals (namodenoson, 100 µg/kg body weight given orally twice daily for 35 days, or control). Tumor width (W) and length (L) were measured twice weekly with a caliber, and tumor size was calculated (W^2^ × L/2).

### 2.4. Statistical Analysis

The inhibition/growth rate (vs. control) was calculated. All data are expressed as mean ± standard error (SE). Analyses were performed with *t*-test and *p*-value < 0.05 was considered statistically significant. The statistical analyses were performed using Excel (Microsoft 365).

## 3. Results

### 3.1. Namodenoson Inhibited Tumor Growth In Vitro in an A_3_AR-Mediated Manner

**Figure 1:**
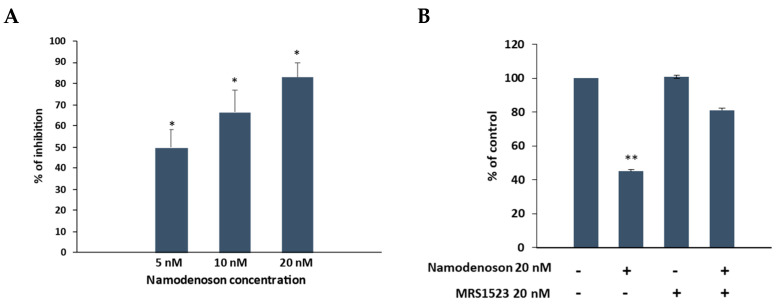
Growth of BxPC-3 cells was inhibited by namodenoson (**A**), and this inhibition was diminished by the A_3_AR antagonist MRS1523 (**B**). Each datapoint represents the mean of 6 and 3 independent experiments for panels (**A**) and (**B**), respectively. The error bars represent SE. * *p* < 0.001, ** *p* < 0.01 (*t*-test vs. control).

In vitro analysis using the Presto Blue assay demonstrated a significant dose-dependent inhibition of BxPC-3 cell growth upon treatment with namodenoson (inhibition of 49.7% ± 8.2%, 66.3% ± 10.5% and 82.7% ± 7.1% for 5 nM, 10 nM, and 20 nM namodenoson, respectively, *p* < 0.001 each; Figure 1A).

Adding the A_3_AR antagonist MRS1523 to the cells (with/without namodenoson) and assessing cell growth using the Presto Blue assay demonstrated that the namodenoson inhibitory effect was A_3_AR-mediated, since treatment with 20 nM MRS1523 had no effect on cell growth (after incubation of 24 h, the growth was 100.8% ± 11.1% of the control), treatment with 20 nM namodenoson had an inhibitory effect (cell growth was 45.0% ± 4.2% of the control), and adding MRS1523 to namodenoson diminished the namodenoson inhibitory effect (cell growth was 81.1% ± 6.3% of the control) (Figure 1B).

### 3.2. Namodenoson Inhibited Tumor Growth In Vivo

**Figure 2:**
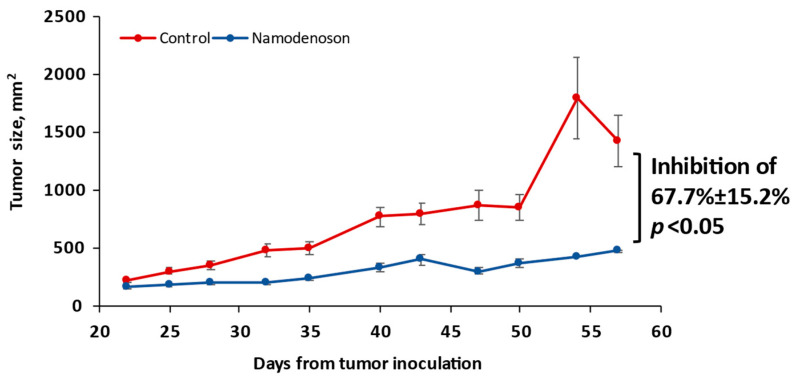
Inhibition of BxPC-3 cell growth by namodenoson in nude mice. Namodenoson was given for 35 days (from Day 22 to Day 57 post tumor inoculation). Each datapoint represents the mean of 10 mice and error bars represent the corresponding SE.

Analysis of the effect of namodenoson (10 µg/kg) given twice daily to nude mice inoculated with BxPC-3 cells for a total of 35 days (from Day 22 to Day 57 post tumor inoculation) demonstrated a significant inhibitory effect of namodenoson on tumor growth (inhibition of 67.7% ± 15.2% by Day 57 from tumor inoculation vs. control, *p* < 0.05; Figure 2).

### 3.3. In Vitro Effects of Namodenoson on Signal Transduction

**Figure 3:**
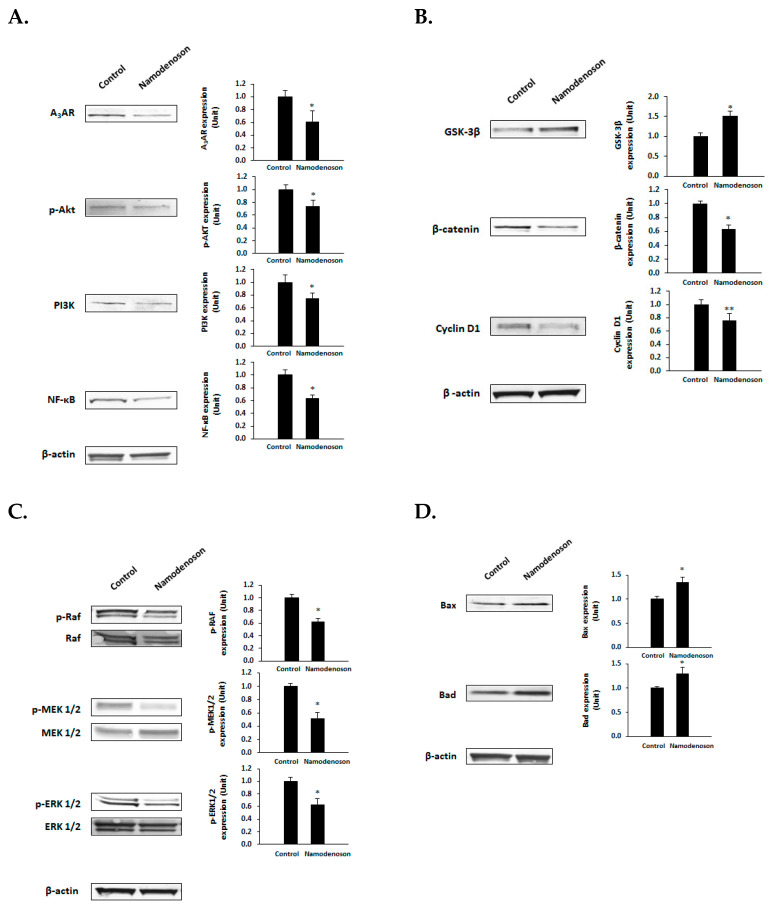
Western blot analysis results of BxPC-3 cells treated with namodenoson vs. controls. (**A**) cell growth regulatory proteins downstream of A_3_AR; (**B**) Wnt/β-catenin signaling pathway proteins; (**C**) RAS downstream proteins; and (**D**) apoptotic proteins. The error bars represent SE. * *p* < 0.05, ** *p* < 0.01 (*t*-test vs. control). Western blot original images can be found in Supplementary Materials.

Western blot analyses using BxPC-3 cells showed statistically significant differences in the expression of regulatory proteins upon treatment with namodenoson. Specifically, namodenoson induced a decrease in A_3_AR protein expression level, and the downstream regulatory proteins p-Akt, PI3K, and NF-κB were all downregulated (Figure 3A). Moreover, the analysis of proteins within the Wnt signal transduction pathway revealed the upregulation of GSK-3β and a decrease in the expression levels of β-catenin and cyclin D1 (Figure 3B). A decrease in the expression levels of proteins downstream from the RAS signaling pathway (pRaf, pMEK 1/2, and pERK 1/2) was also observed (Figure 3C). In addition, the two apoptotic proteins Bad and Bax were upregulated, suggesting that the apoptosis of the BxPC-3 cells was induced (Figure 3D).

## 4. Discussion

The current study demonstrated that namodenoson inhibited the growth of pancreatic adenocarcinoma in vitro and in vivo in a mechanism that was A_3_AR-mediated and involved the deregulation of the NFκB, Wnt/β-catenin, and the RAS signaling pathways leading to induction of apoptosis.

Our in vitro and in vivo findings are consistent with former studies investigating namodenoson in liver cancer, in which A_3_AR-mediated growth inhibition was observed and the mechanism-of-action was shown to involve the NF-κB as well as the Wnt pathways and apoptosis induction [13,14,15]. The mechanism of action described in the present work is also aligned with former studies demonstrating anti-tumor effects in melanoma, colon, prostate, lung, and thyroid carcinoma, as well as lymphoma. The molecular mechanism of action included the deregulation of proteins downstream from A_3_AR activation (p-Akt, PI3K, and NF-κB) and the upregulation of GSK-3β, which led to the down-regulation of β-catenin and subsequent decrease in cyclin D1, resulting in the upregulation of apoptotic proteins [16,17]. These effects were all induced by nanomolar concentrations of the agonists, which selectively targeted A_3_AR and not other adenosine receptor family members which may confer opposite effects. Moreover, namodenoson was found to potentiate the activity of natural killer (NK) cells by inducing IL-12 production, a cytotoxic factor with antitumor effects [18].

The findings in this study are also consistent with the established role of the Wnt and RAS pathways in pancreatic cancer [3,5]. Indeed, targeting the Wnt and RAS pathways as therapeutic approaches in pancreatic cancer has been attempted, albeit with limited success. Targeting the Wnt signaling pathway is challenging, as this pathway is shared by normal and cancer cells, leading to significant treatment-associated toxicities. For example, vantictumab, a monoclonal antibody that interacts with five frizzled receptors and blocks their interactions with the Wnt ligand, led to significant adverse events, including gastrointestinal and fragility fractures [19]. Targeting the RAS pathway and specifically KRAS is a challenge due to the KRAS structure (i.e., lack of pharmacologically targetable pockets within KRAS mutants) and development of tumor resistance. Recent improvements in drug design have led to the clinical development of effective inhibitors against cancers with the mutant KRAS^G12C^, two of which (sotorasib and adagrasib) have been approved by the Food and Drug Administration for previously treated advanced-stage KRAS^G12C^-mutant non-small cell lung cancer (NSCLC) [20,21,22].

KRAS mutant tumors have been shown to resist farnesyl transferases inhibitor (FTI) therapy. FTIs inhibit the post-translational prenylation of KRAS (required for its membrane localization and hence its signal transduction), and resistance occurs due to alternative prenylation [23,24].

Our findings that namodenoson inhibited pancreatic tumor growth, combined with the excellent safety profile of namodenoson, as shown in clinical trials in HCC, as well as in other indications such as non-alcoholic fatty liver disease [10,11,25], support further preclinical research on namodenoson in pancreatic cancer and its continued development as a potential treatment for this difficult-to-treat disease.

## 5. Conclusions

Our preclinical findings support the continued development of namodenoson as a treatment for pancreatic cancer.

## Data Availability

Data supporting this study are included within the article.

## Supplementary Materials

The following supporting information can be downloaded at: https://www.mdpi.com/article/10.3390/biom13111584/s1.
