# Supplementary figures and images for: Namodenoson Inhibits the Growth of Pancreatic Carcinoma via Deregulation of the Wnt/β-catenin, NF-κB, and RAS Signaling Pathways
Source: Biomolecules. 2023 Oct 27;13(11):1584. doi: 10.3390/biom13111584 (PMC10669398; doi:10.3390/biom13111584)

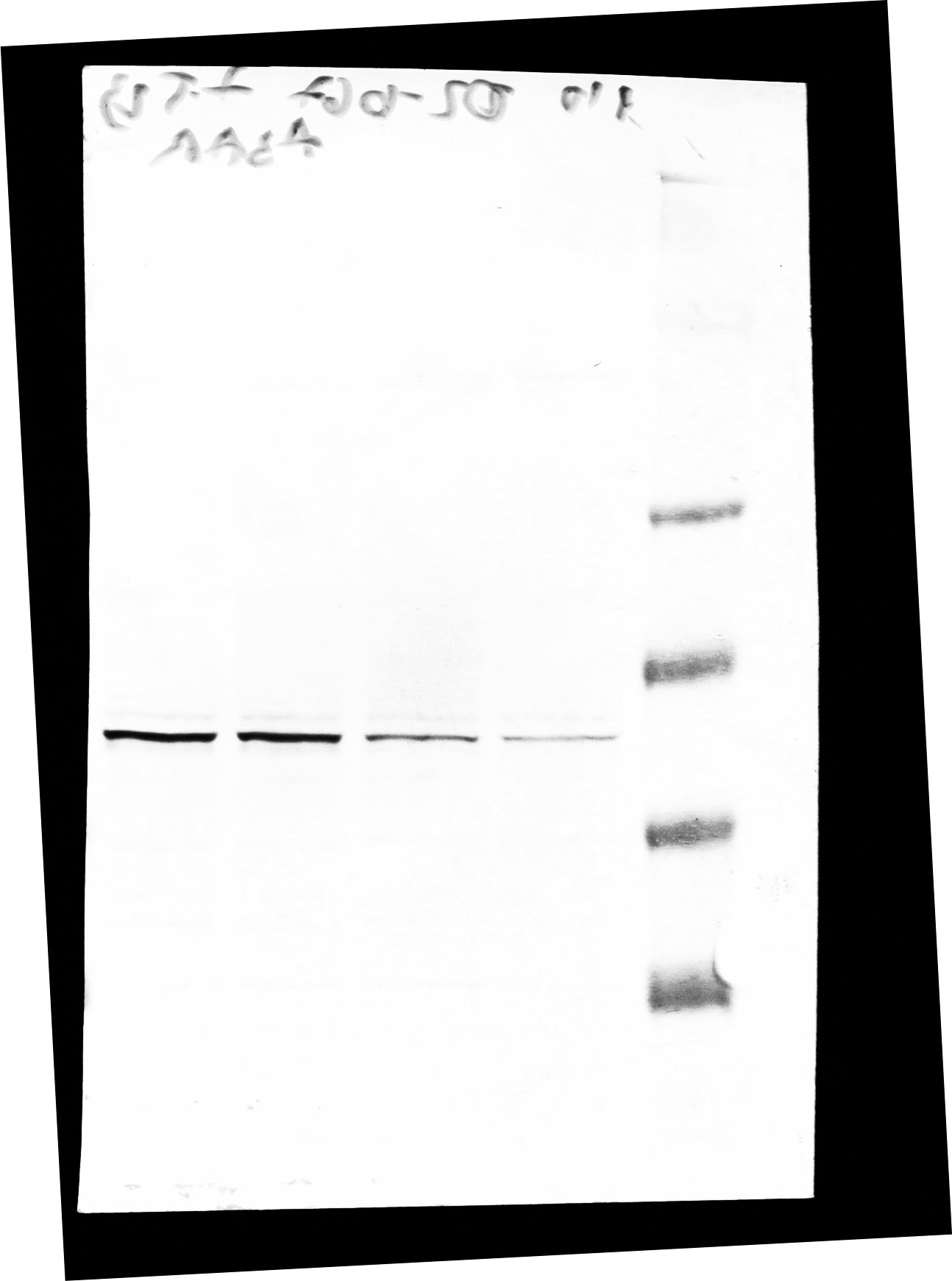

Supplement: Supplementary file 1 [file biomolecules-13-01584-s001.zip › 3A-A3AR.jpg]

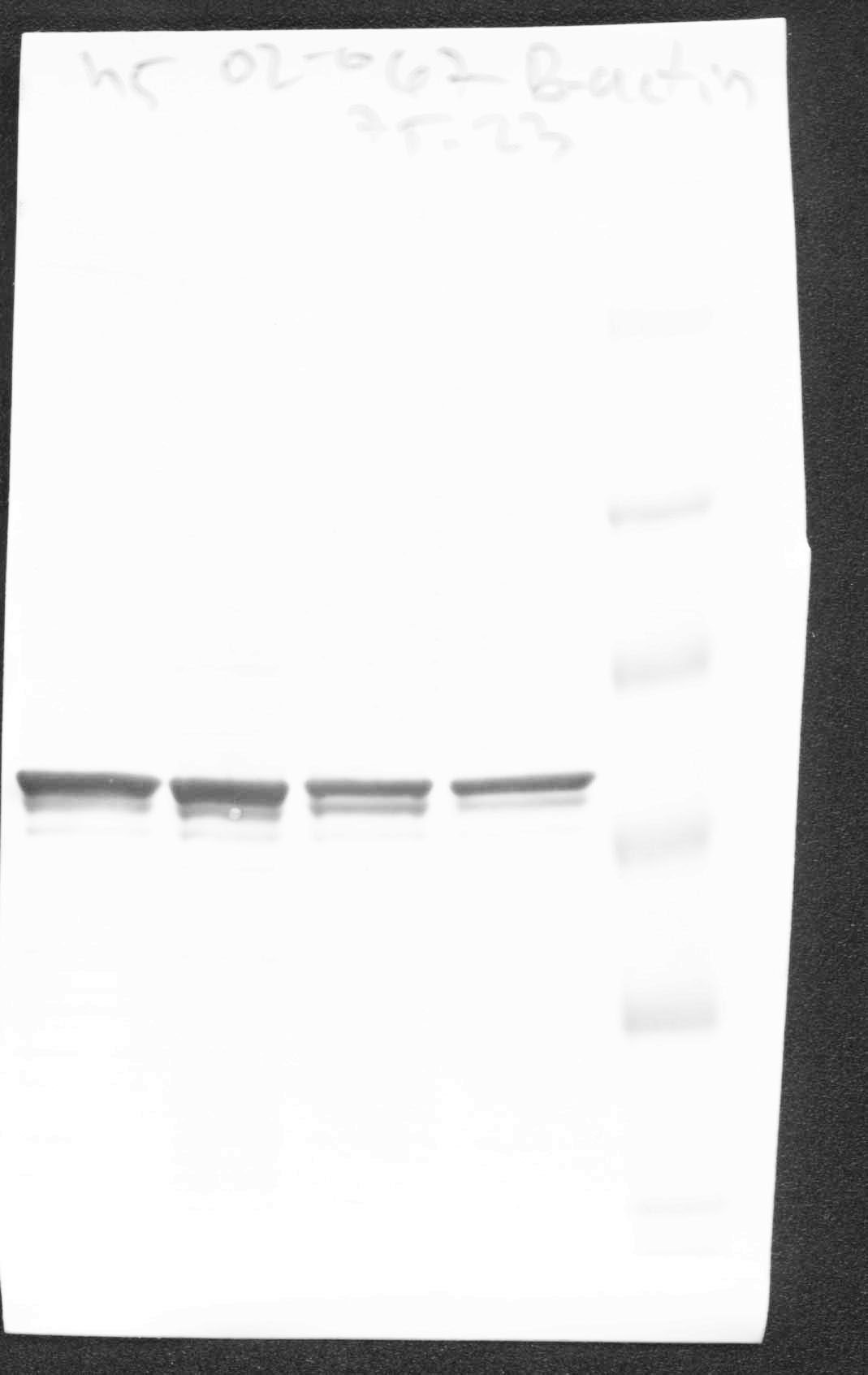

Supplement: Supplementary file 1 [file biomolecules-13-01584-s001.zip › 3A-beta-actin.jpg]

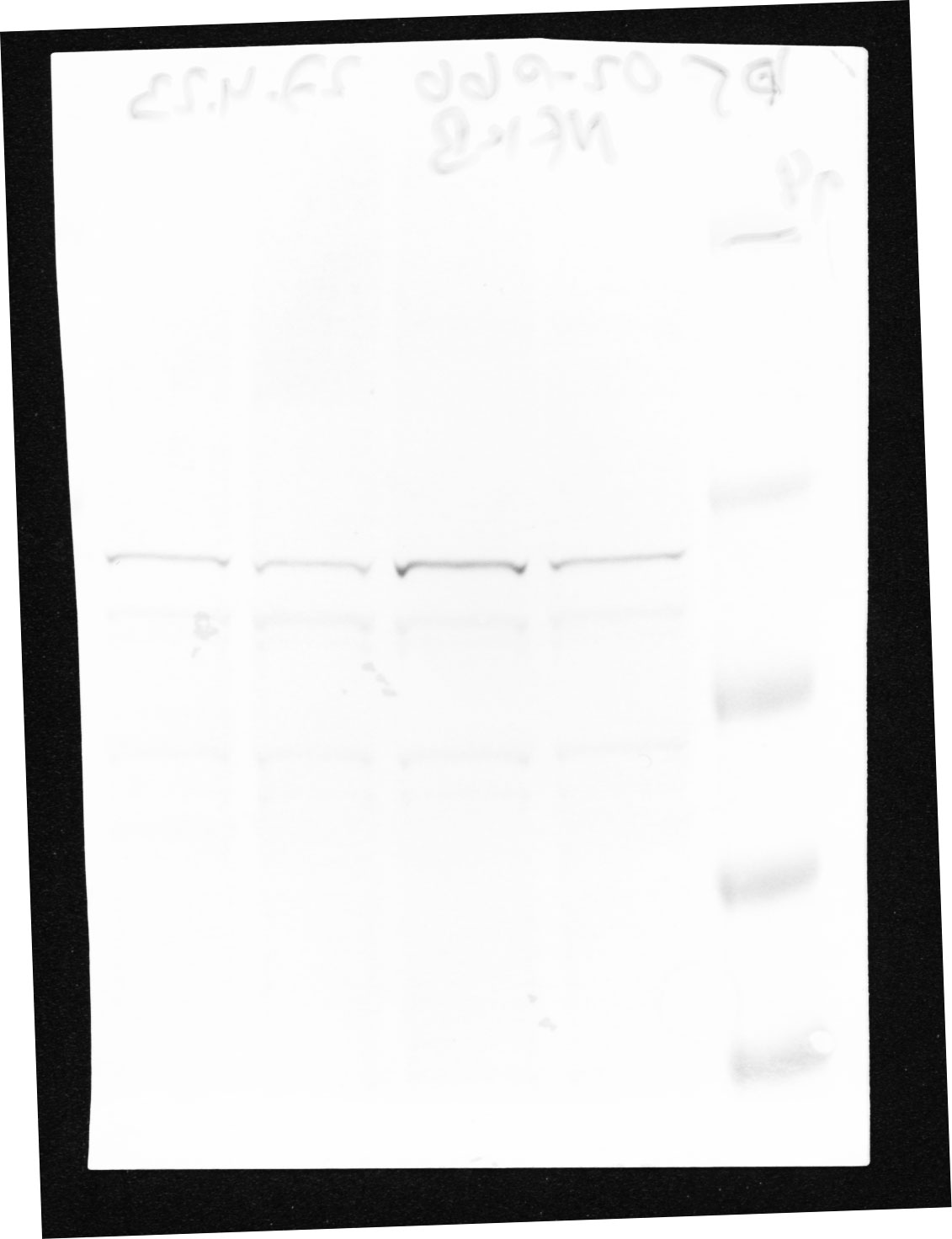

Supplement: Supplementary file 1 [file biomolecules-13-01584-s001.zip › 3A-NFKB.jpg]

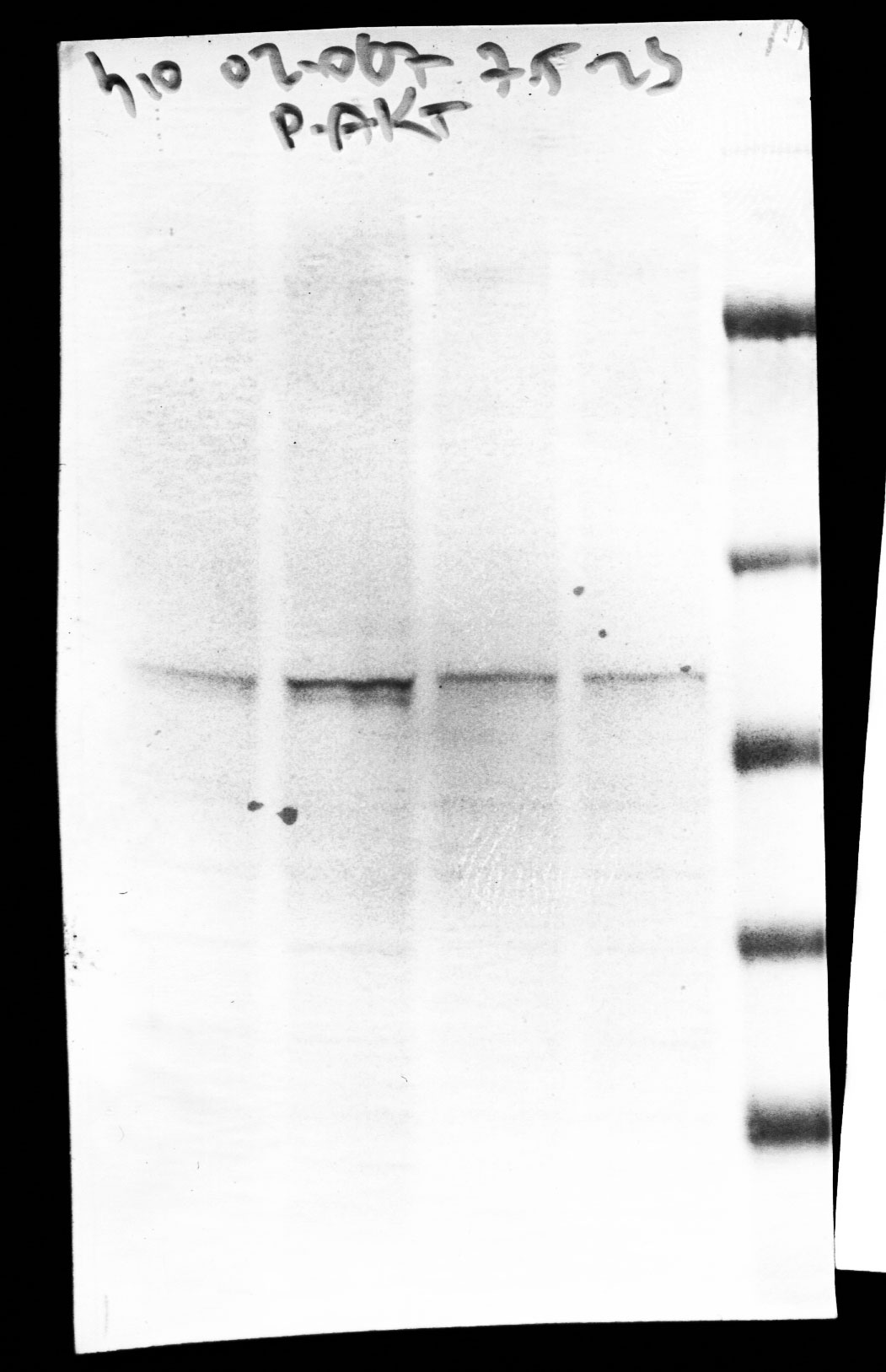

Supplement: Supplementary file 1 [file biomolecules-13-01584-s001.zip › 3A-P-AKT.jpg]

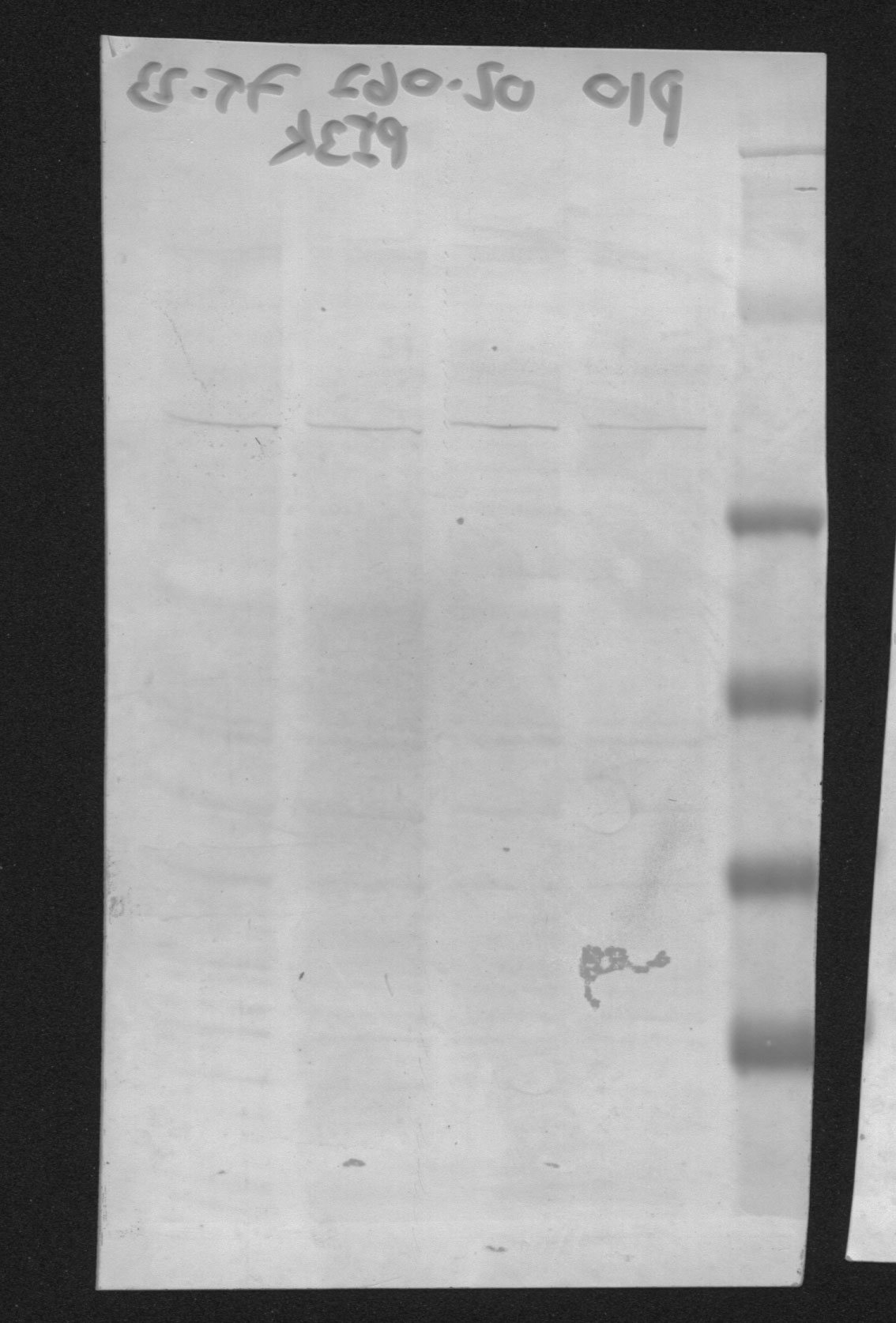

Supplement: Supplementary file 1 [file biomolecules-13-01584-s001.zip › 3A-PI3K.jpg]

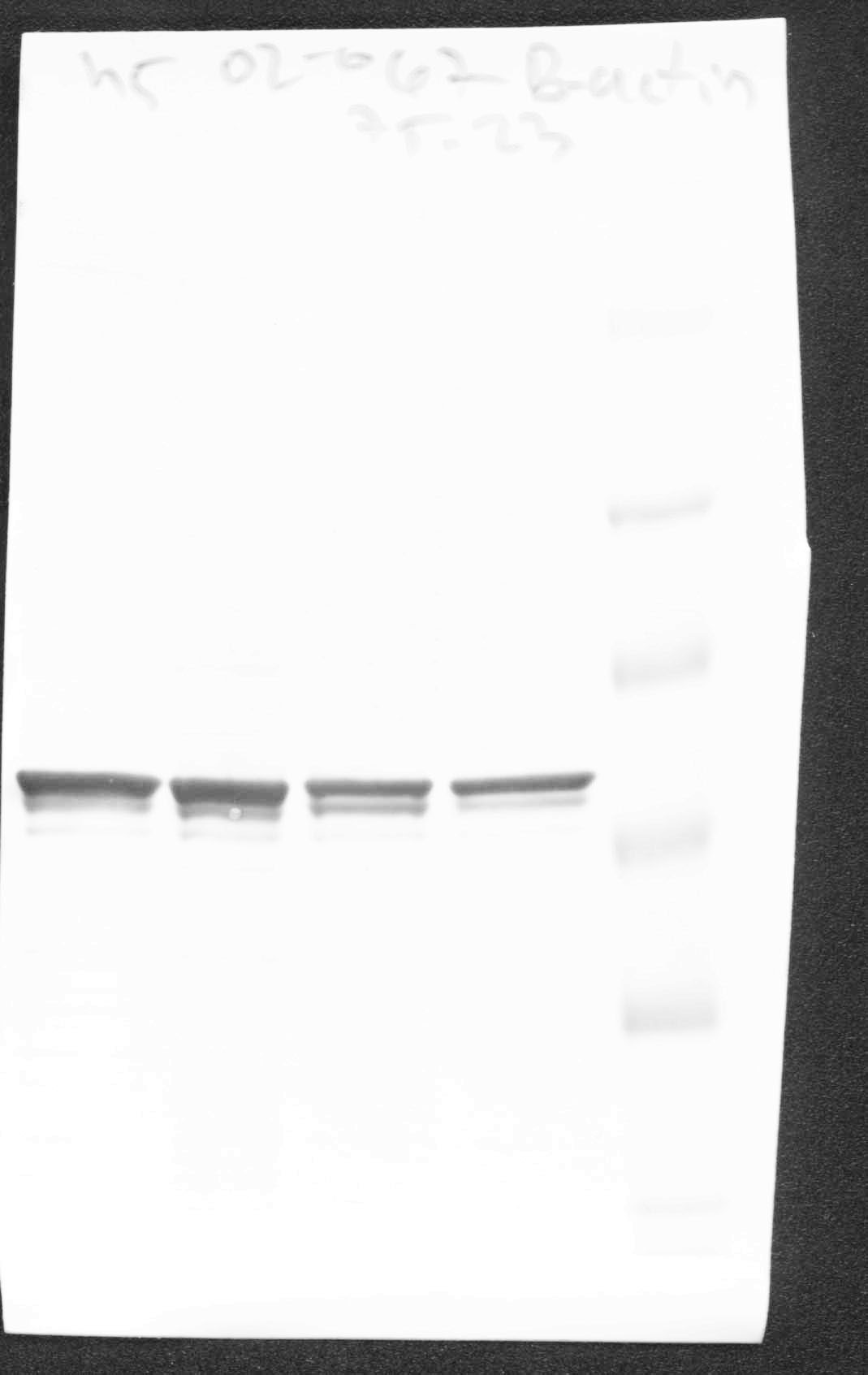

Supplement: Supplementary file 1 [file biomolecules-13-01584-s001.zip › 3B-beta-actin.jpg]

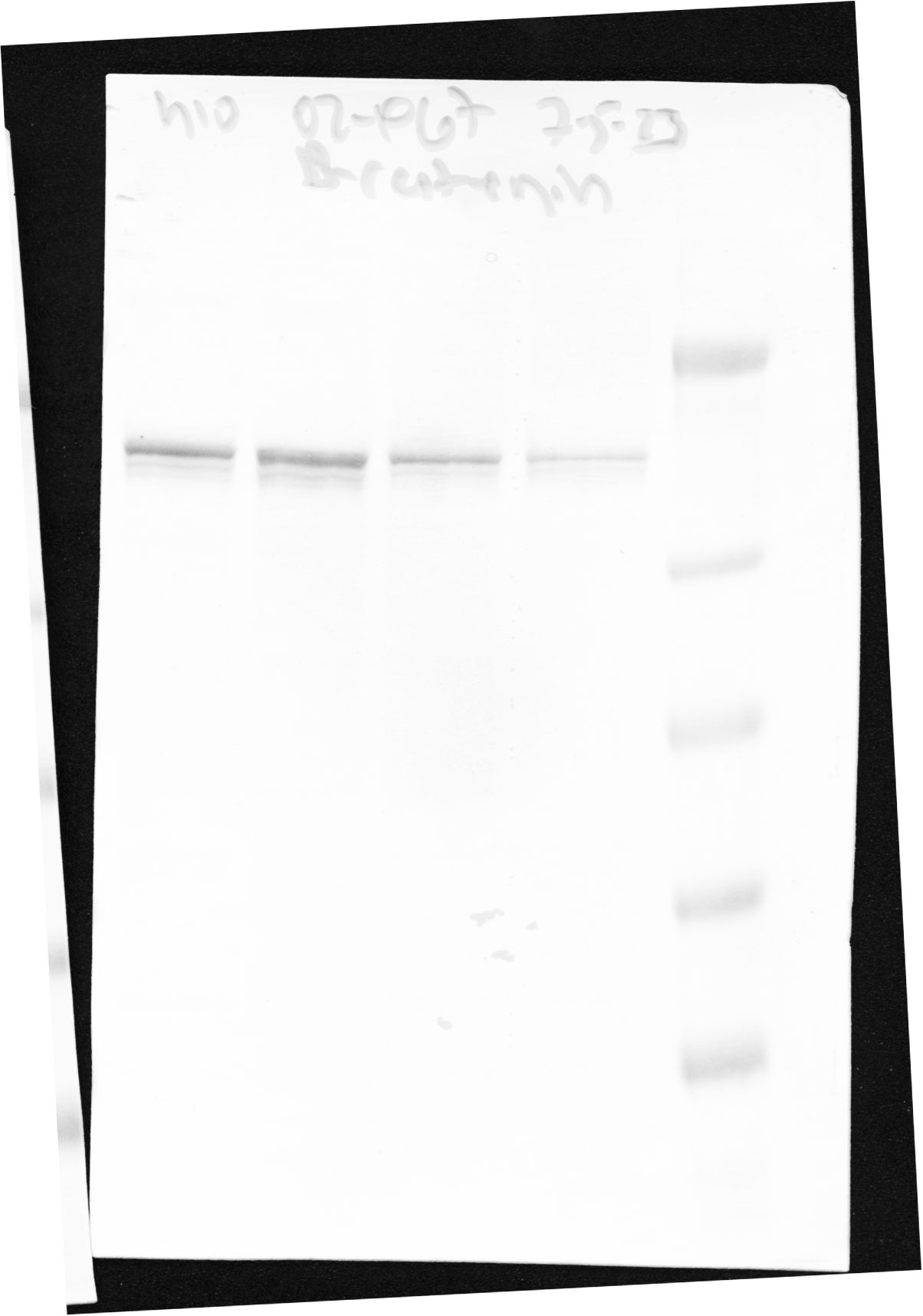

Supplement: Supplementary file 1 [file biomolecules-13-01584-s001.zip › 3B-beta-catenin.jpg]

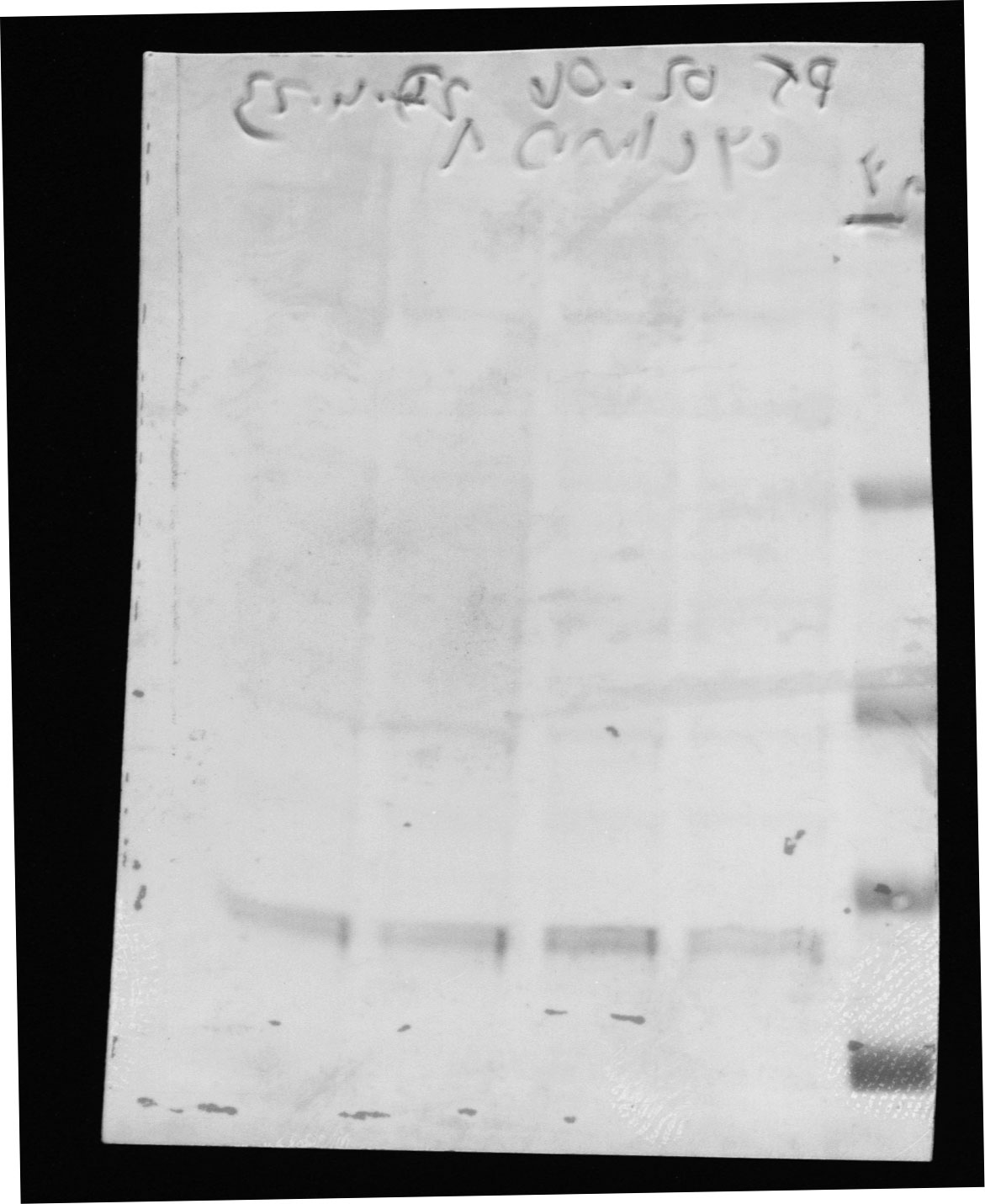

Supplement: Supplementary file 1 [file biomolecules-13-01584-s001.zip › 3B-cyclin-D1.jpg]

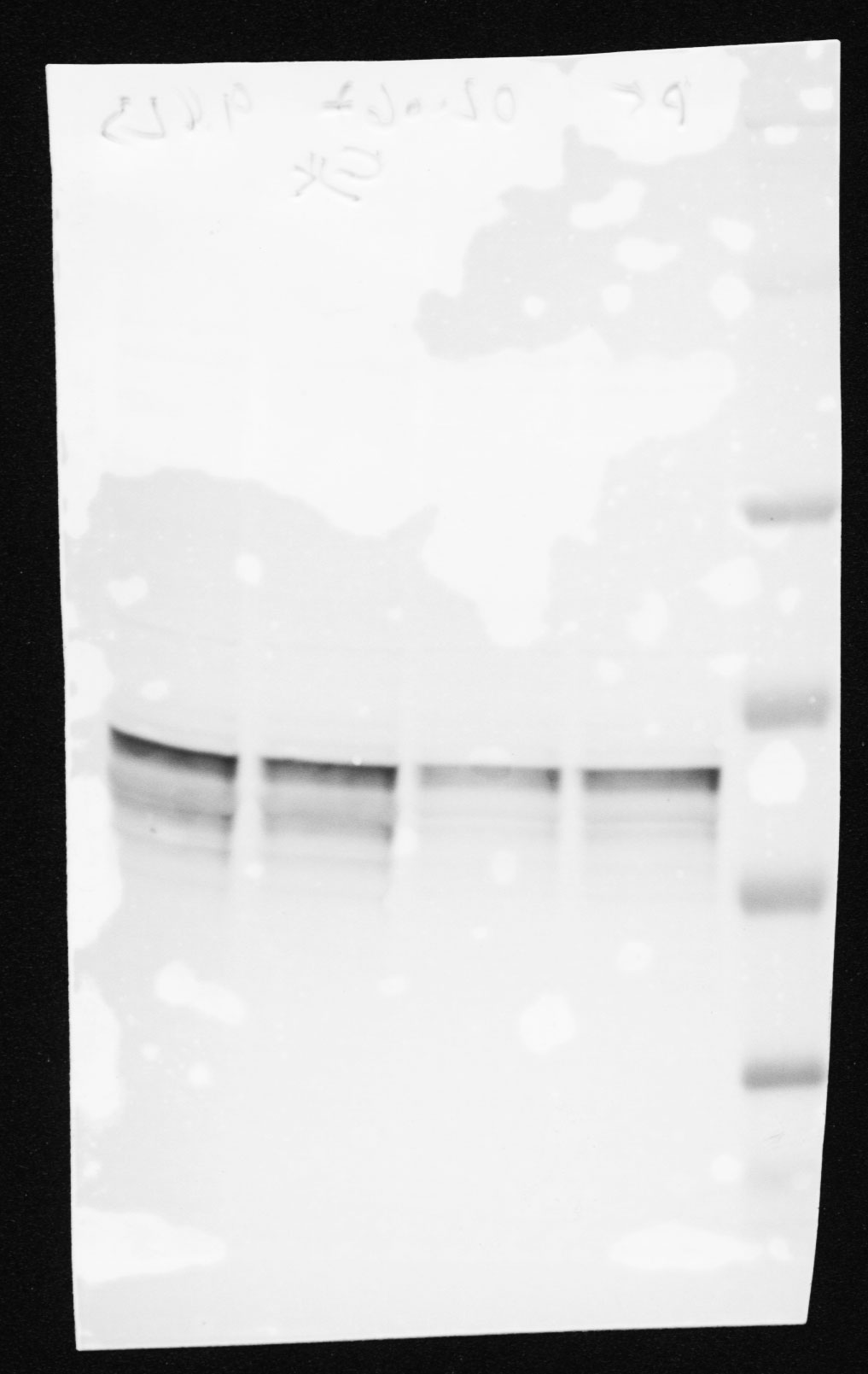

Supplement: Supplementary file 1 [file biomolecules-13-01584-s001.zip › 3B-GSK3beta.jpg]

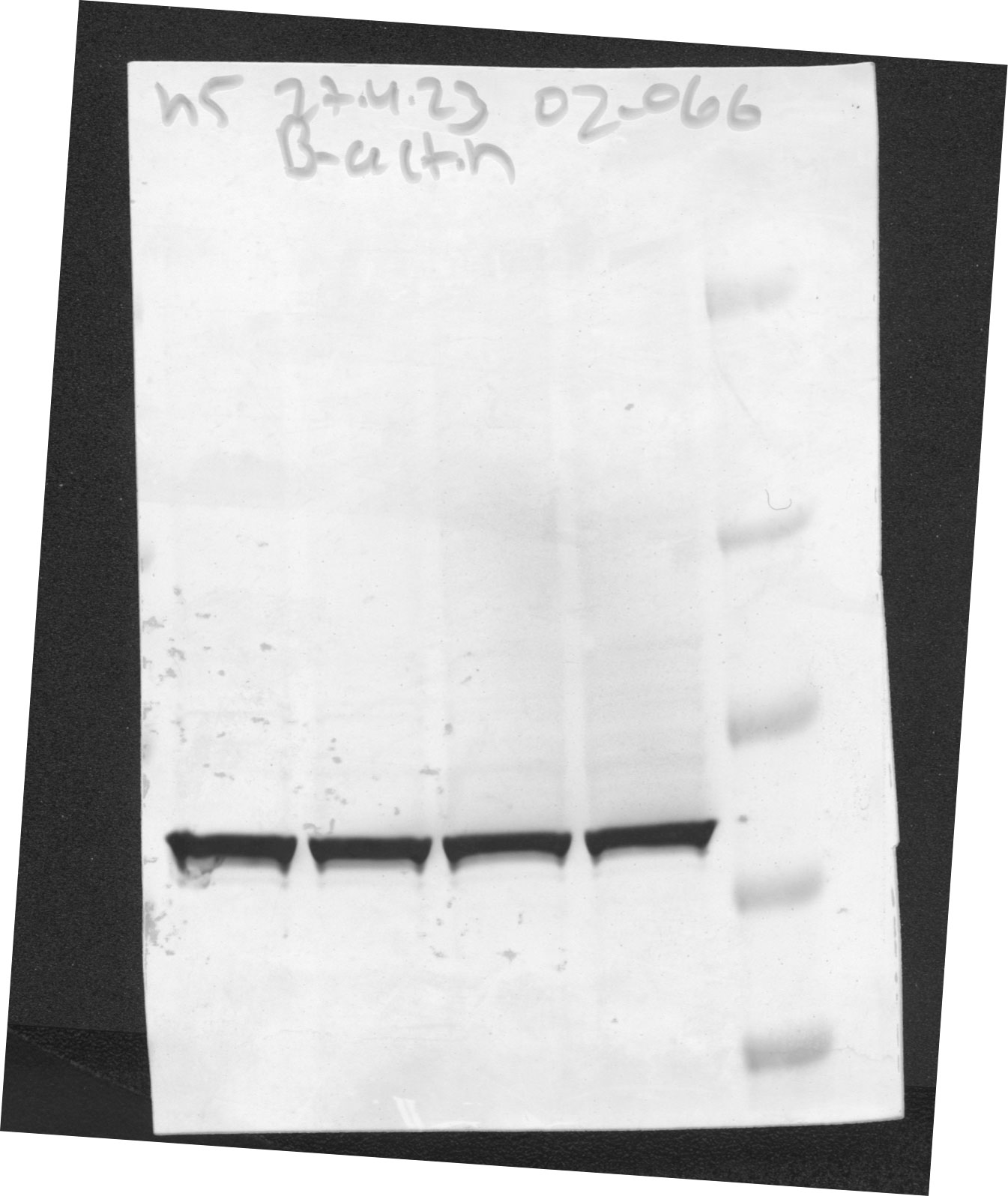

Supplement: Supplementary file 1 [file biomolecules-13-01584-s001.zip › 3C-beta-actin.jpg]

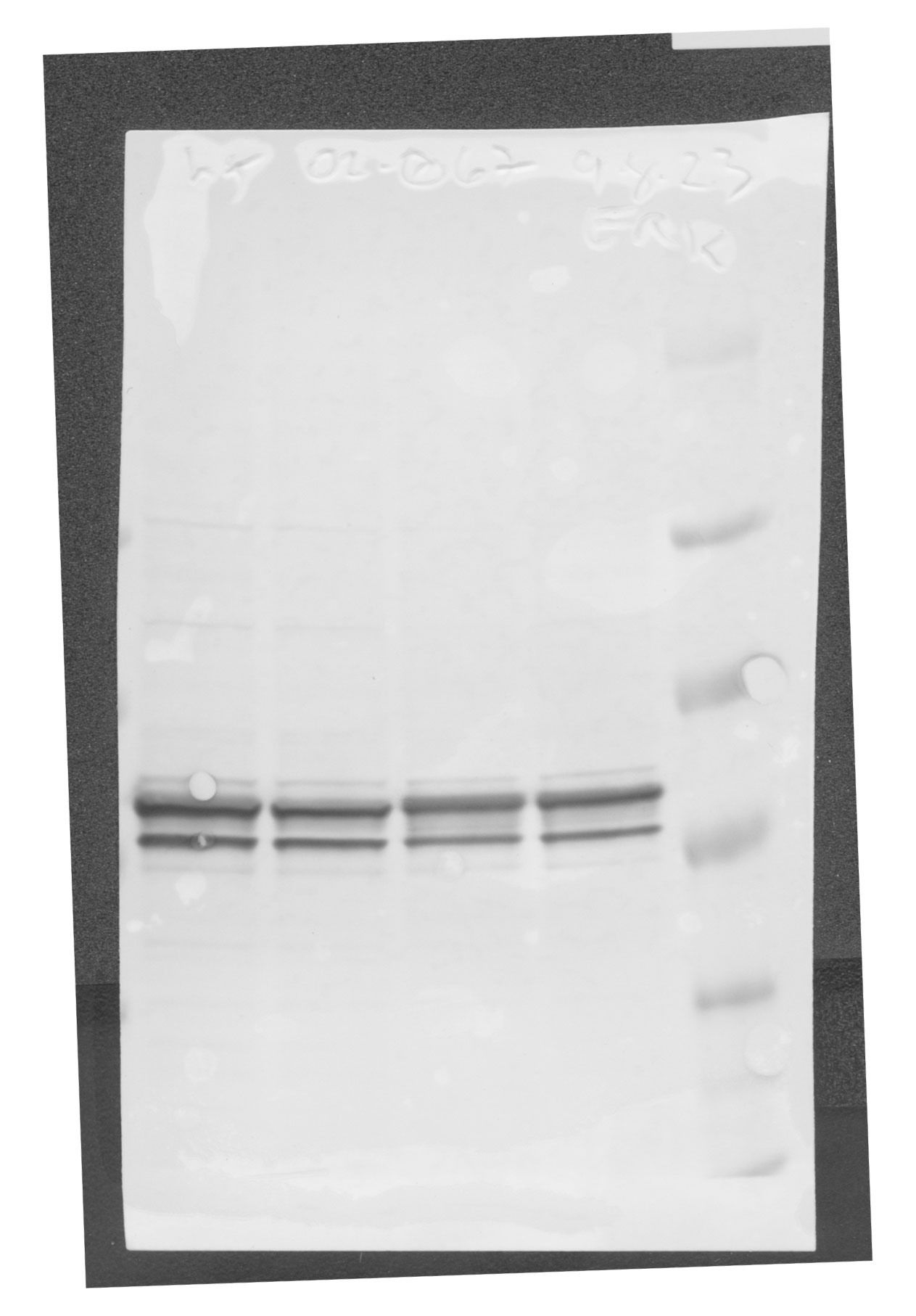

Supplement: Supplementary file 1 [file biomolecules-13-01584-s001.zip › 3C-ERK1-2.jpg]

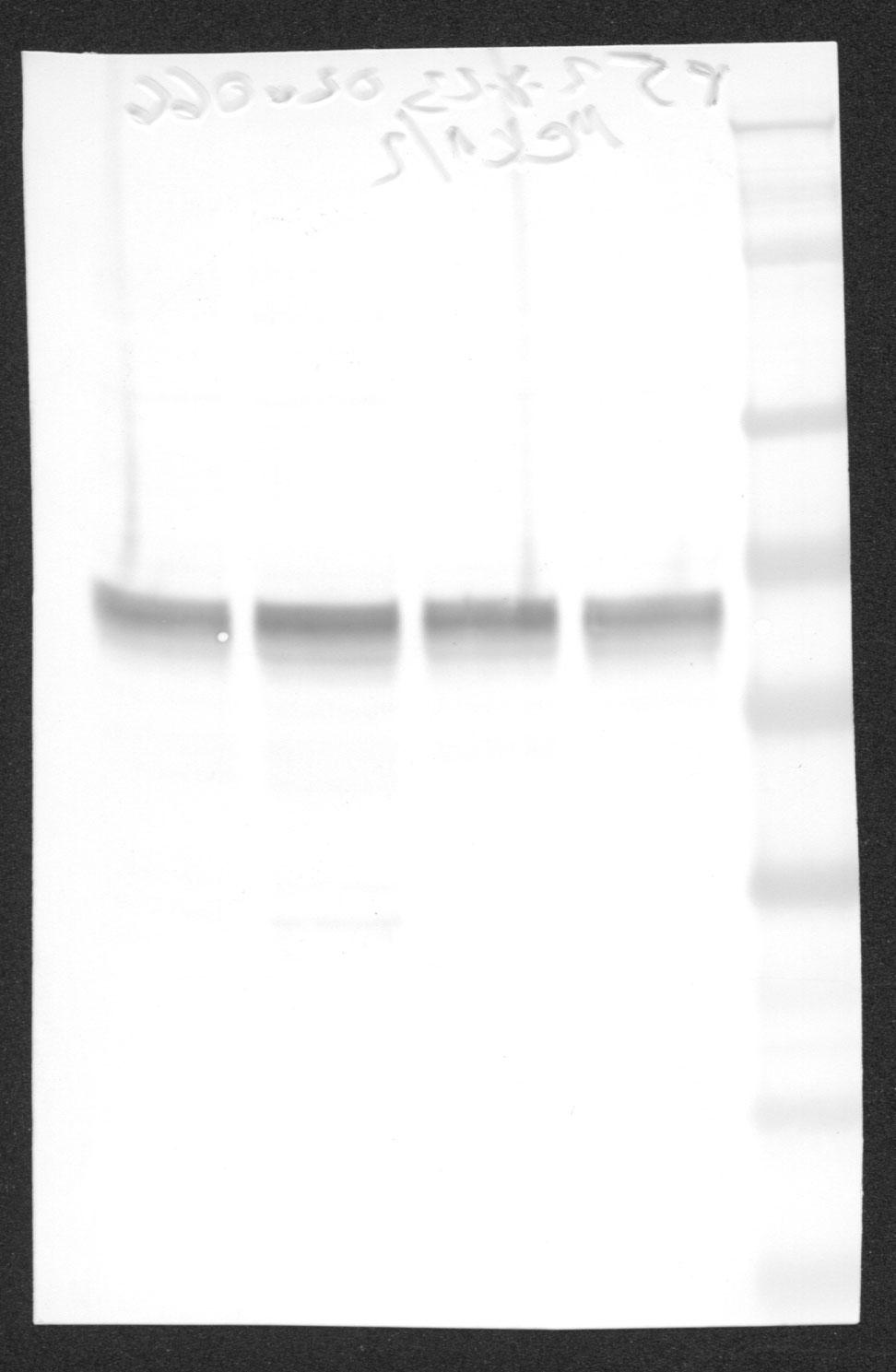

Supplement: Supplementary file 1 [file biomolecules-13-01584-s001.zip › 3C-MEK1-2.jpg]

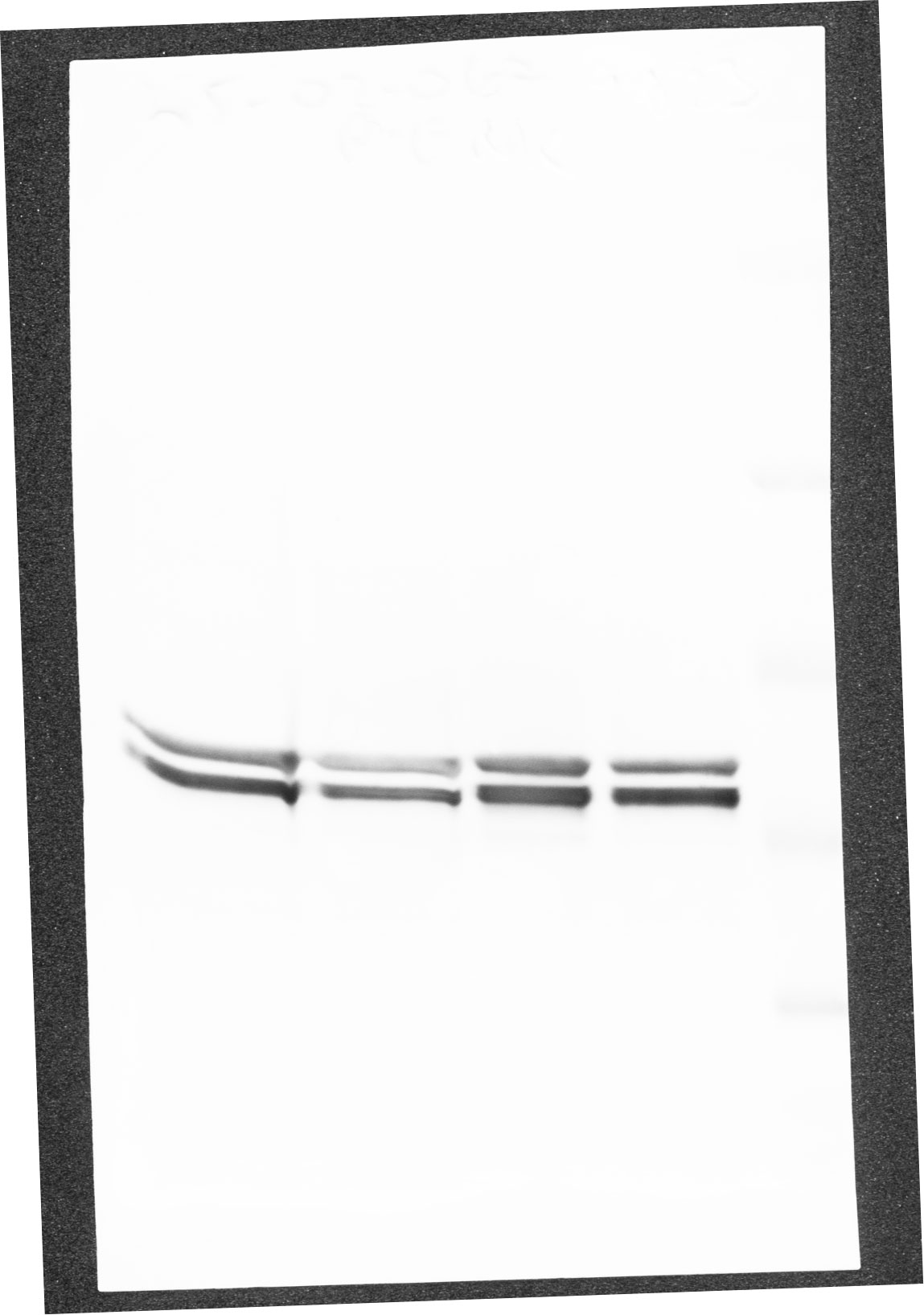

Supplement: Supplementary file 1 [file biomolecules-13-01584-s001.zip › 3C-p-ERK1-2.jpg]

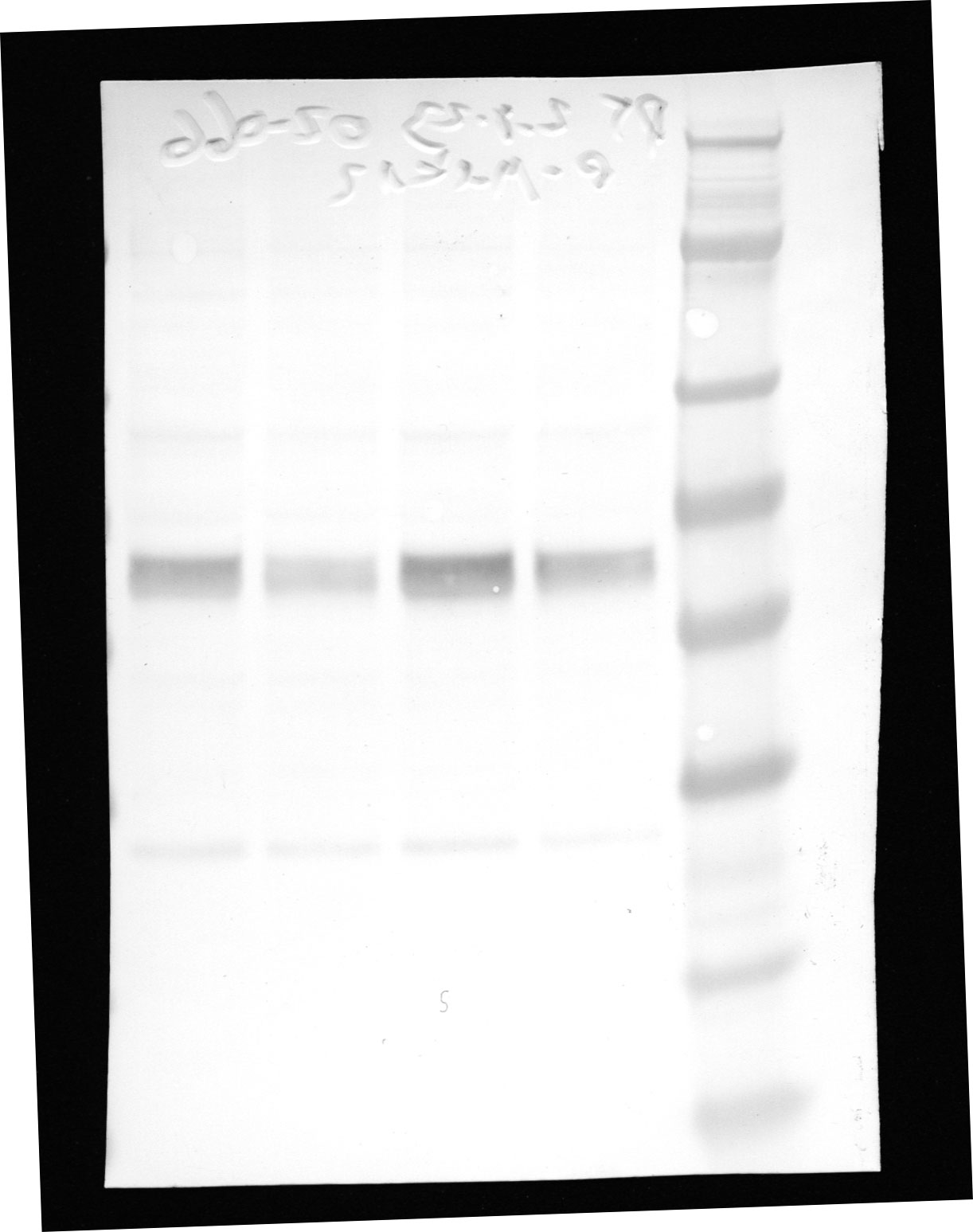

Supplement: Supplementary file 1 [file biomolecules-13-01584-s001.zip › 3C-p-MEK1-2.jpg]

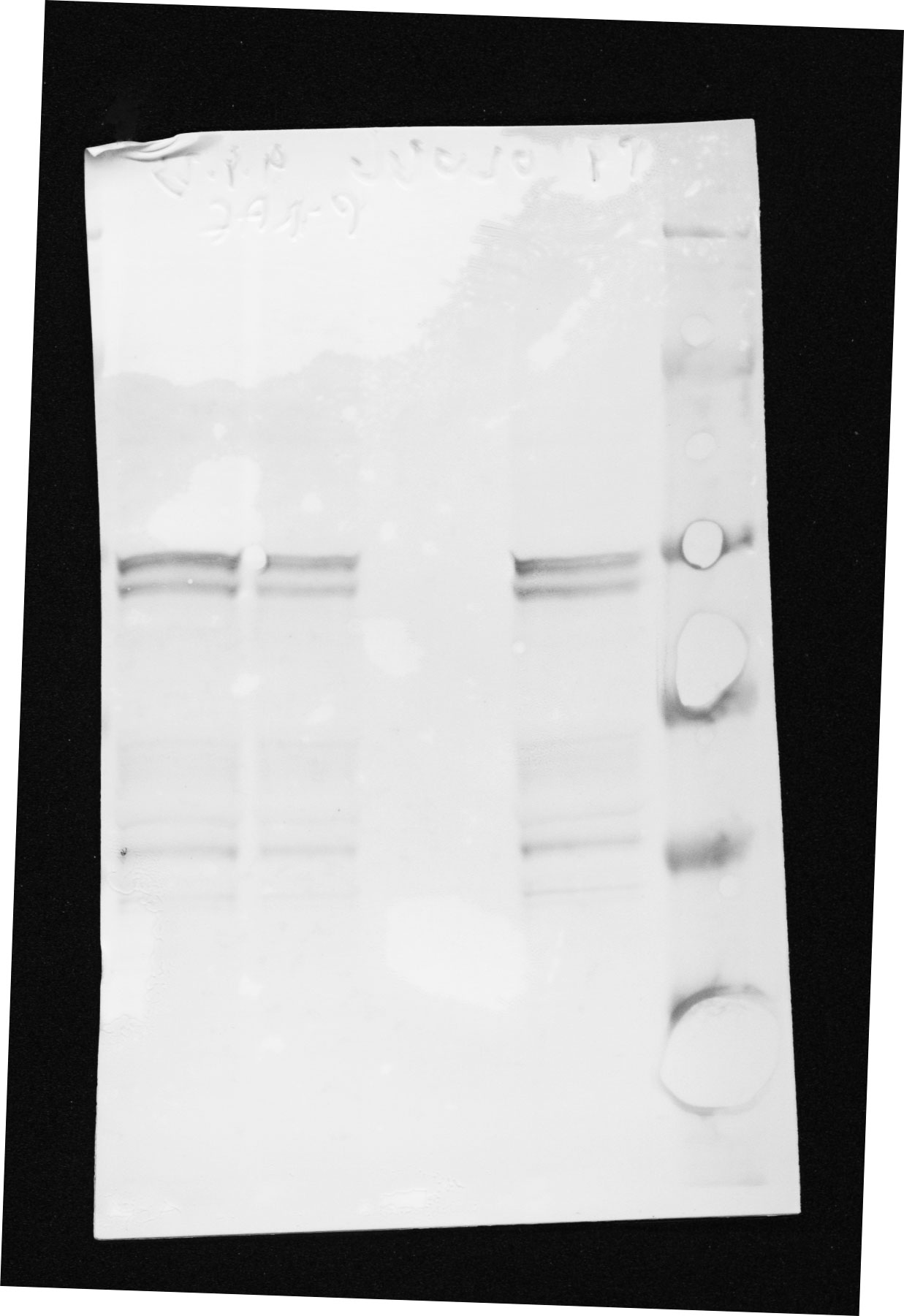

Supplement: Supplementary file 1 [file biomolecules-13-01584-s001.zip › 3C-p-Raf.jpg]

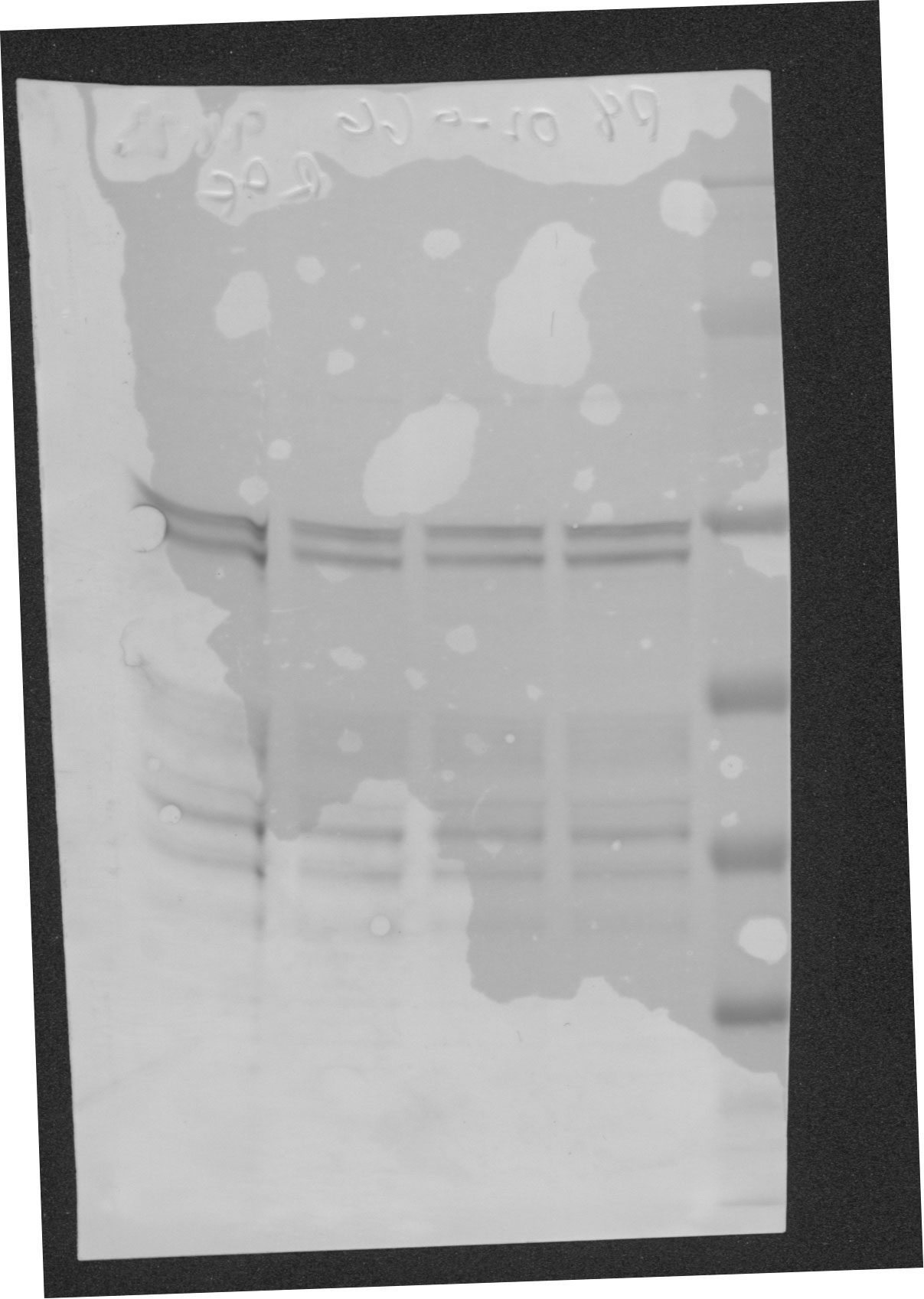

Supplement: Supplementary file 1 [file biomolecules-13-01584-s001.zip › 3C-RAF.jpg]

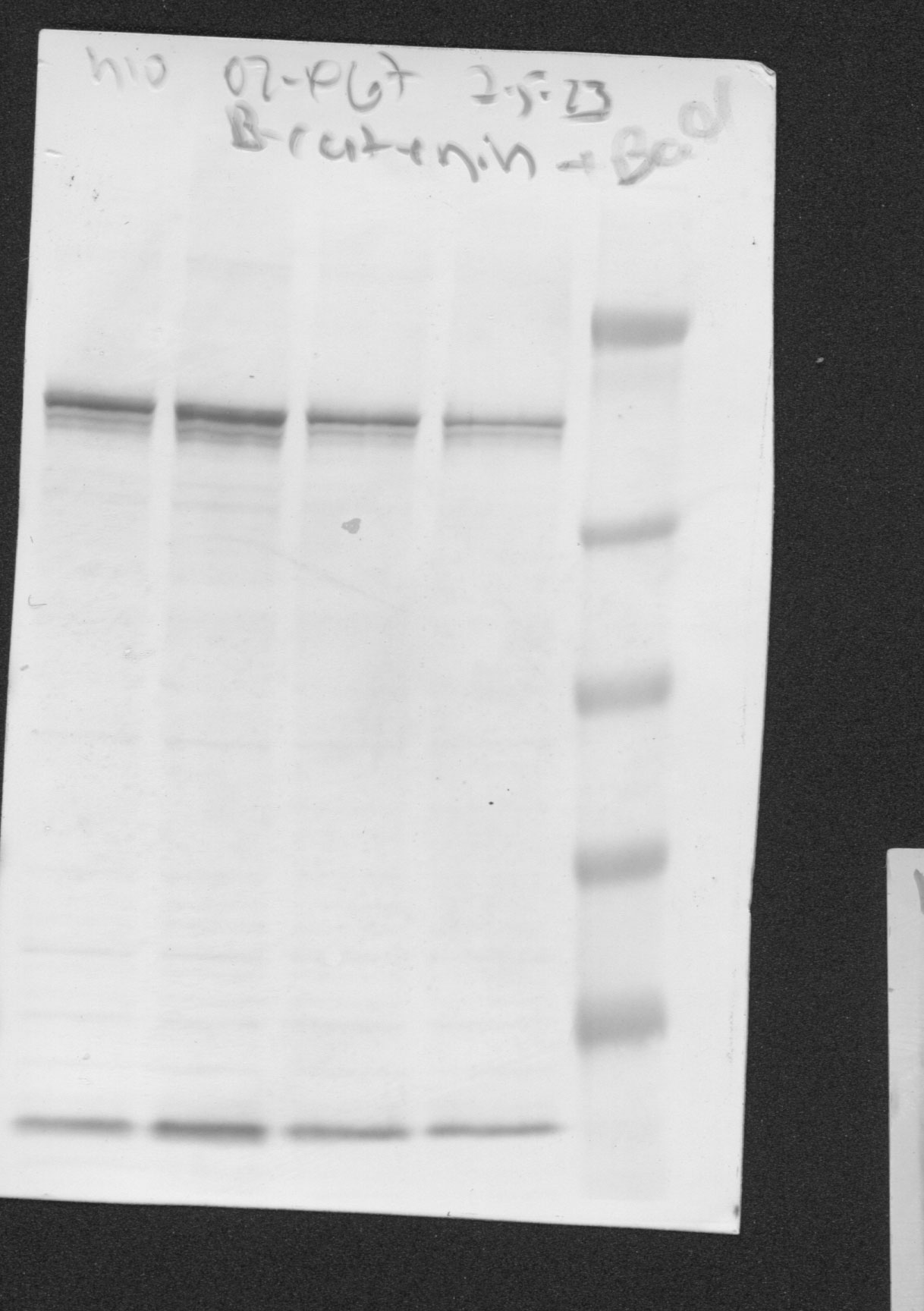

Supplement: Supplementary file 1 [file biomolecules-13-01584-s001.zip › 3D-Bad.jpg]

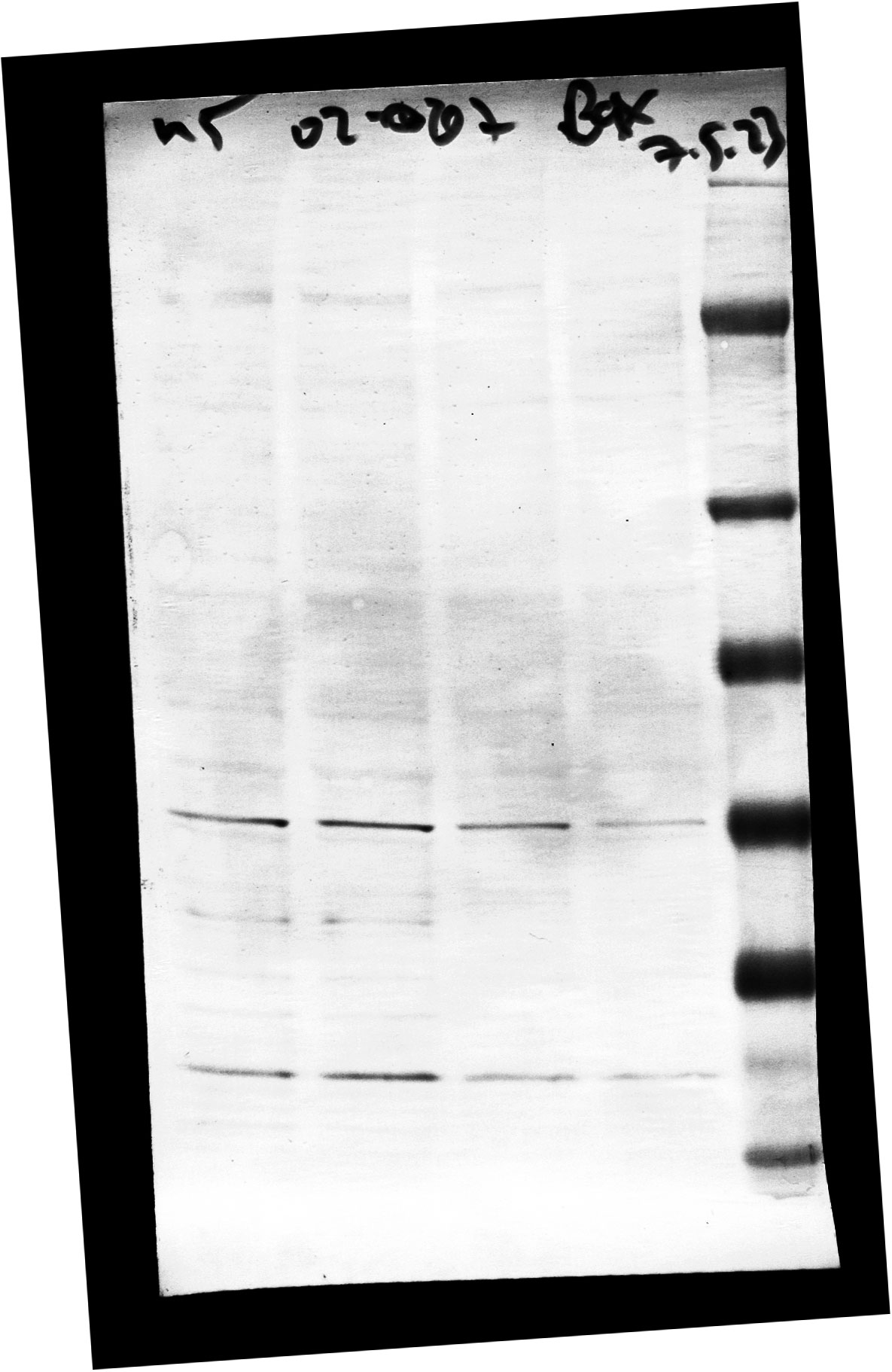

Supplement: Supplementary file 1 [file biomolecules-13-01584-s001.zip › 3D-Bax.jpg]

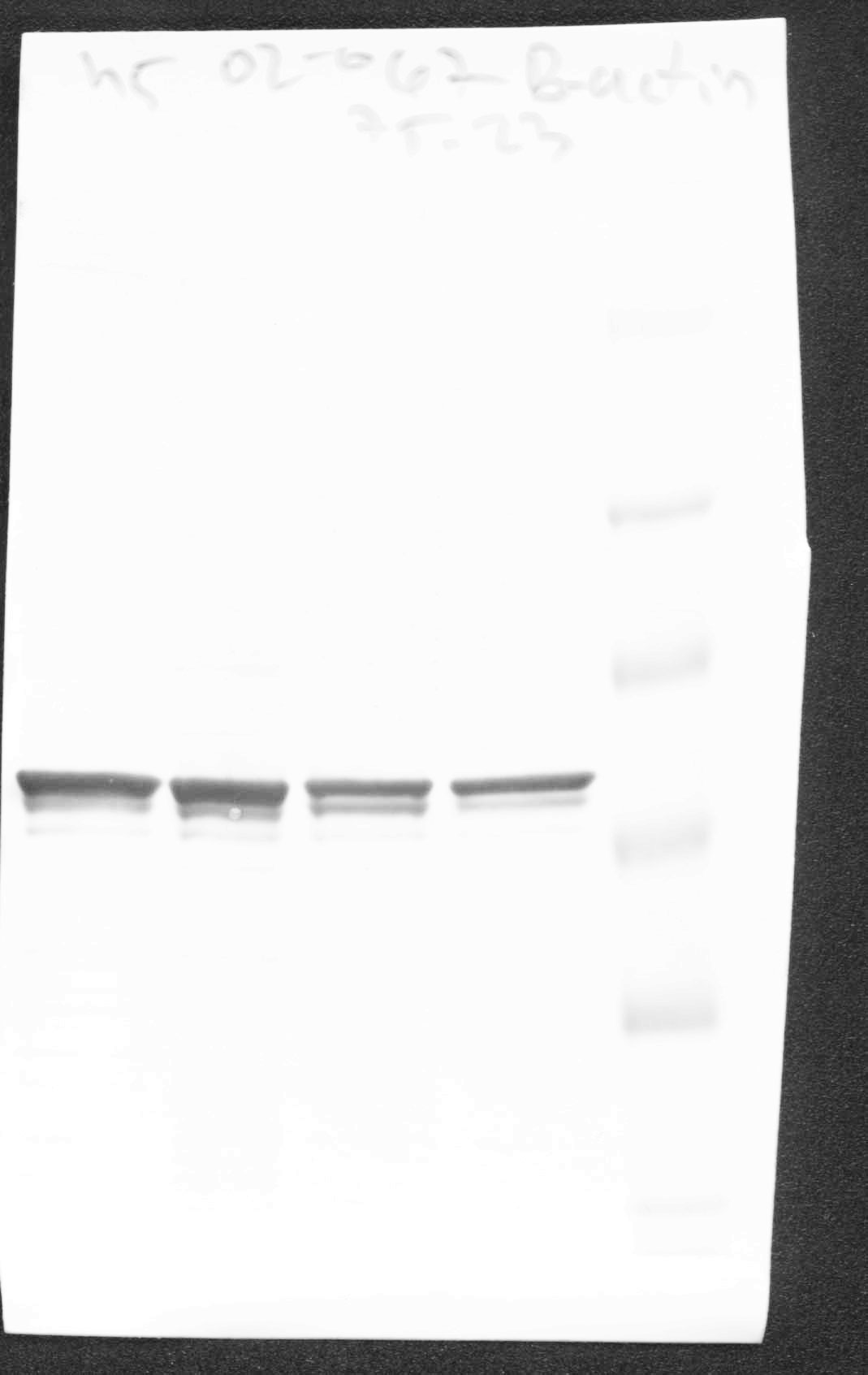

Supplement: Supplementary file 1 [file biomolecules-13-01584-s001.zip › 3D-beta-actin.jpg]
